# P-930. A Retrospective Cohort Study on the Impact of Prophylactic Antibiotics on Surgical Site Infections in Gastrointestinal Surgery During a Cefazolin Shortage

**DOI:** 10.1093/ofid/ofaf695.1134

**Published:** 2026-01-11

**Authors:** Hiroya Kojima, Hiroaki Hata, Kohei Fujita

**Affiliations:** National Hospital Organization Kyoto Medical Center, Kyoto City, Kyoto, Japan; Kyoto Medical Center, Kyoto, Kyoto, Japan; National Hospital Organization Kyoto Medical Center, Kyoto City, Kyoto, Japan

## Abstract

**Background:**

During cefazolin (CEZ) shortages, alternative antibiotics with broader antimicrobial spectra were recommended for surgical prophylaxis. Given the global emphasis on combating antimicrobial resistance (AMR), it is critical to avoid unnecessary broad-spectrum antibiotic use. This shortage period offered a unique opportunity to evaluate the effectiveness of broader-spectrum antibiotics, typically not recommended prophylactically, compared to CEZ in preventing surgical site infections (SSIs) in clean-contaminated surgeries.

**Methods:**

We retrospectively assessed SSI incidence among patients undergoing elective cholecystectomy, gastrectomy, esophagectomy, or hepatectomy in which CEZ was used as the standard prophylactic antibiotic from February 2018 to June 2020. Primary outcomes included SSI incidence; secondary outcomes were rates of multidrug-resistant organism (MDRO) detection and *Clostridioides difficile* infection (CDI). SSI diagnoses followed CDC/NHSN criteria.

**Results:**

The study included 2,290 patients (1,326 males, 964 females; median age 68 years, median BMI 23.2 kg/m²). SSI rates were similar between CEZ (5.8%, 77/1,330) and broad-spectrum antibiotic groups (5.0%, 48/960; OR 1.17, p=0.412). MDRO detection rates showed no significant difference (CEZ 5.2%, broad-spectrum 6.2%; OR 0.83, p=0.811). CDI incidence was 0.1% overall, with no significant difference between groups (OR 1.45, p=0.764).

**Conclusion:**

No significant differences were observed between CEZ and broad-spectrum antibiotic groups regarding SSI incidence, MDRO detection, or CDI rates. This is the first direct comparison of CEZ versus alternative antibiotics for prophylaxis in clean-contaminated surgeries, providing valuable clinical and economic insights. During CEZ shortages, broad-spectrum antibiotics did not demonstrate superiority over CEZ in preventing SSIs, reinforcing recommendations against their routine prophylactic use.

**Disclosures:**

All Authors: No reported disclosures

